# Liver Lipidomics Analysis Revealed the Novel Ameliorative Mechanisms of L-Carnitine on High-Fat Diet-Induced NAFLD Mice

**DOI:** 10.3390/nu15061359

**Published:** 2023-03-10

**Authors:** Chengyuan Sun, Yan Guo, Peixu Cong, Yuan Tian, Xiang Gao

**Affiliations:** 1 College of Life Sciences, Qingdao University, Qingdao 266071, China; 2 Xiangyang Central Hospital, Affiliated Hospital of Hubei University of Arts and Science, Xiangyang 441021, China; 3 College of Food Science and Engineering, Ocean University of China, Qingdao 266100, China

**Keywords:** L-carnitine, non-alcoholic fatty liver disease, inflammation, lipid metabolism, lipidomics

## Abstract

The beneficial effects of L-carnitine on non-alcoholic fatty liver disease (NAFLD) were revealed in previous reports. However, the underlying mechanisms remain unclear. In this study, we established a high fat diet (HFD)-induced NAFLD mice model and systematically explored the effects and mechanisms of dietary L-carnitine supplementation (0.2% to 4%) on NAFLD. A lipidomics approach was conducted to identify specific lipid species involved in the ameliorative roles of L-carnitine in NAFLD. Compared with a normal control group, the body weight, liver weight, concentrations of TG in the liver and serum AST and ALT levels were dramatically increased by HFD feeding (*p* < 0.05), accompanied with obvious liver damage and the activation of the hepatic TLR4/NF-κB/NLRP3 inflammatory pathway. L-carnitine treatment significantly improved these phenomena and exhibited a clear dose–response relationship. The results of a liver lipidomics analysis showed that a total of 12 classes and 145 lipid species were identified in the livers. Serious disorders in lipid profiles were noticed in the livers of the HFD-fed mice, such as an increased relative abundance of TG and a decreased relative abundance of PC, PE, PI, LPC, LPE, Cer and SM (*p* < 0.05). The relative contents of PC and PI were significantly increased and that of DG were decreased after the 4% L-carnitine intervention (*p* < 0.05). Moreover, we identified 47 important differential lipid species that notably separated the experimental groups based on VIP ≥ 1 and *p* < 0.05. The results of a pathway analysis showed that L-carnitine inhibited the glycerolipid metabolism pathway and activated the pathways of alpha-linolenic acid metabolism, glycerophospholipid metabolism, sphingolipid metabolism and Glycosylphosphatidylinositol (GPI)-anchor biosynthesis. This study provides novel insights into the mechanisms of L-carnitine in attenuating NAFLD.

## 1. Introduction

Non-alcoholic fatty liver disease (NAFLD) is a manifestation of metabolic syndrome in the liver; it ranges from hepatic steatosis to non-alcoholic steatohepatitis (NASH), and it can further develop into fibrosis and cirrhosis [1]. Currently, approximately 25% of the global population suffers from NAFLD [2]. As reported, the prevalence of NAFLD in China has increased from 18% to 29% in a decade [3,4]. NAFLD is closely related to many metabolic diseases, such as type 2 diabetes (T2D) and cardiovascular disease (CVD) [1]. For instance, NAFLD is highly prevalent (above 75%) in patients with T2D [5]. The risk of CVD increases one-fold in people with NAFLD [6]. To date, no pharmacological treatment for NAFLD is available. Currently, the mainstay of NAFLD prevention and therapy is still lifestyle intervention, including a healthy diet and physical exercise. However, this strategy has the limitation of poor compliance [7,8]. Hence, the dietary supplementation of nutrients or natural food ingredients has become an important approach for NAFLD therapy.

L-carnitine is a vitamin-like substrate, which transports fatty acids into the mitochondria for β-oxidation. An increasing number of studies have indicated that L-carnitine might be beneficial for the treatment of NAFLD. In high fat diet (HFD)-induced obese mice, 500 mg/kg L-carnitine oral gavage for 25 weeks significantly prevented an increase in triglycerides (TGs) and cholesterol (TC) in the liver [9]. In NAFLD mice induced by methionine and choline deficiency, L-carnitine (200 mg/kg) supplementation for 3 weeks notably reduced hepatic lipid accumulation [10]. In humans, patients with NAFLD displayed decreased serum levels of L-carnitine [11]. A recent randomized controlled trial-based meta-analysis proved that L-carnitine supplementation effectively reduced the serum levels of aspartate transaminase (AST), alanine transaminase (ALT) and TG and the homeostasis model assessment of insulin resistance (HOMA-IR) in patients with NAFLD [12]. However, the mechanisms underlying the protective effect of L-carnitine on NAFLD are not fully understood. The promotion of fatty acid β-oxidation through the “carnitine shuttle” explains part of the lipid clearing effect of L-carnitine in the liver [13]. In addition, Su et al. indicated that L-carnitine ameliorated hepatic steatosis by downregulating the gene expressions of lipogenesis and upregulating the gene expressions of cholesterol clearance/catabolism and energy expenditure [9]. Other studies also attribute the protective effect of L-carnitine on NAFLD to its antioxidant and anti-inflammation properties [14,15]. Nowadays, traditional clinical techniques are insufficient to characterize the pathogenesis of NAFLD [16]. Thus, omics technology emerged to help researchers to elucidate the mechanisms of NAFLD and identify the metabolic pathways underlying the improvement effects of dietary supplements.

Lipidomics, a novel omics strategy, is a sensitive and effective approach for the determination of lipid composition and unique lipid species associated with various metabolic diseases, such as NAFLD [17,18]. Previous lipidomics studies revealed that NAFLD is associated with an altered hepatic lipid profile, such as increased diacylglycerol (DAG), altered fatty acid composition, increased free cholesterol (FC) and decreased phosphatidylcholine (PC), in the liver of humans and rodents [19,20]. In HFD-induced NAFLD mice, a lipidomics approach revealed that phytosterols ameliorated NAFLD by decreasing TGs with a higher number of polyunsaturated fatty acids in the liver [21]. Liu et al. reported that cereal β-glucan modulated hepatic glycerophospholipids, glycerolipids and sphingolipid metabolism to ameliorate NAFLD in Western diet-induced NAFLD mice through a UPLC/MS-based lipidomics analysis [22]. To date, no study has used lipidomics to explore the efficacy of L-carnitine in attenuating NAFLD.

In this study, we aimed to determine the specific hepatic lipid profiles associated with the NAFLD-alleviating effects of L-carnitine based on a lipidomics analysis in HFD-fed mice. The findings provide novel insights into the mechanism of L-carnitine in preventing NAFLD.

## 2. Materials and Methods

### 2.1. Animals and Diets

The animal study was approved by the Animal Ethics Committee of Qingdao University (No. 20210310C576J800728013) [23]. Male C57BL/6J mice of 6 weeks old (licensed ID: SCXK2019-0010) were provided by SPF Biotechnology Co., Ltd. (Beijing, China). After a 1-week adaptation period, the mice were separated into 2 groups: a control group (Con, *n* = 10), fed with a normal chow diet, and a group fed an HFD with 60% energy from fat (HF, *n* = 60). After 8 weeks, the mice in the HF group were randomly assigned into six groups: one HF group and five L-carnitine intervention groups, LC1, LC2, LC3, LC4 and LC5, that were fed with an HFD containing 0.2%, 0.5%, 1%, 2% and 4% L-carnitine, respectively.

### 2.2. Sample Collection

After 10 weeks of feeding, the 12 h fasted mice were sacrificed to separate serum. Fresh liver tissue samples were isolated for histopathology determinations or quick-frozen with liquid nitrogen and stored until further analyses, such as a biochemical indices analysis, a histopathological analysis, Western blot and a lipidomics study.

### 2.3. Basic Biochemical Measurements

The serum levels of ALT and AST and the hepatic concentrations of TG were measured using commercial kits [24].

The concentrations of TNF-α, IL-6, IL-10 and IL-1β in liver tissue were detected using ELISA kits [23].

### 2.4. Histological Analyses

The frozen sections of livers were stained with Oil Red O. The histological images were observed using a regular light microscope according to a previous report [25].

### 2.5. Western Blot Analysis

The liver tissues were homogenized in a cold RIPA lysis buffer to prepare hepatic homogenate. Equal amounts of protein determined using BCA kits were separated on 10% SDS-PAGE; transferred onto PVDF membranes; blocked with 5% skim milk; and incubated with NLRP3, TLR4, NF-κB, JNK or β-actin primary antibodies (Cell Signaling, Danvers, MA, USA) overnight at 4 °C. Afterwards, the membrane was washed with TBST and incubated with a secondary antibody (Cell Signaling). β-actin was considered the internal control. The bands of protein were observed and quantified using a chemiluminescence (ECL) localization reagent and a Tanon GIS analysis [24].

### 2.6. Lipidomics Analysis

The liver tissue samples (100 mg) were transferred to EP tubes and homogenized in 1 mL 0.9% NaCl. Then, 750 μL IPA(iso-Propyl alcohol) was added to 50 μL of the tissue homogenate solution, followed by ultrasonic extraction for 1 h. Subsequently, the extract was centrifuged at 10,000 rpm for 10 min. Finally, 100 μL of supernatant was loaded into the LC tube and set aside. The lipidomics analysis was performed on a RPLC-Q Exactive-MS/MS system (Thermo Fisher, Waltham, MA, USA) with an Acquity UPLC BEH C18 column (2.1 mm × 100 mm, 1.7 μm) (Waters, Milford, MA, USA) as previously described [26].

### 2.7. Data Processing and Statistical Analysis

All data are presented as mean ± standard deviation (SD). The mean differences among the groups were analyzed with a one-way ANOVA, followed by Tukey’s post hoc test, using SPSS 20.0 (Chicago, IL, USA). GraphPad Prism 8 (San Diego, CA, USA) was used for figure preparation. Differences between groups were considered statistically significant at *p* < 0.05.

## 3. Results

### 3.1. Effects of L-Carnitine on the Growth Parameters of NAFLD Mice

**Figure 1:**
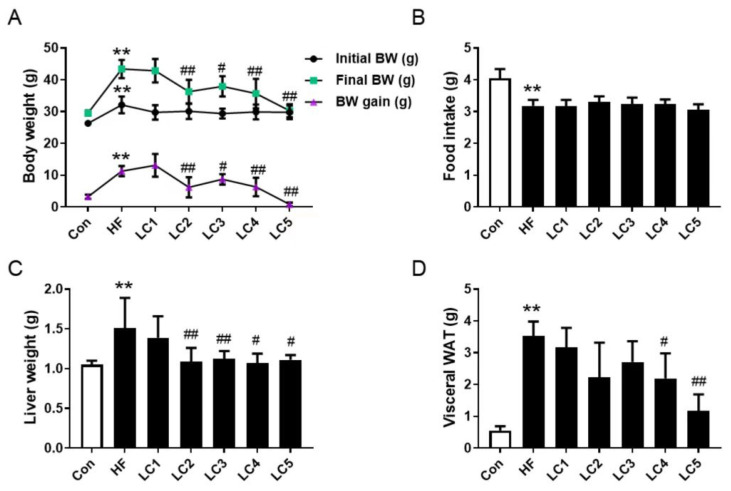
Effects of L-carnitine on growth parameters in HFD-induced NAFLD mice. (**A**) Body weight; (**B**) food intake; (**C**) liver weight; (**D**) visceral WAT: visceral white adipose tissue; *n* = 10 each group; ** *p* < 0.01 to Con; ^#^ *p* < 0.05, ^##^ *p* < 0.01 to HF.

The data of the growth parameters are presented in our previous study [23] and Figure 1. The HF group displayed a dramatic increase in body weight gain, liver weight and visceral white adipose tissue (WAT) compared to the Con group (*p* < 0.01 for all). The LC2-5 groups had a significantly lower body weight gain and liver weight than the HF group (*p* < 0.05 for all). The LC4-5 groups also showed a significant decline in WAT (*p* < 0.05 for all). The food intake of the HF group was lower than that of the Con group (*p* < 0.01).

### 3.2. Effect of L-Carnitine on Hepatic Lipid Accumulation and Liver Function in NAFLD Mice

**Figure 2:**
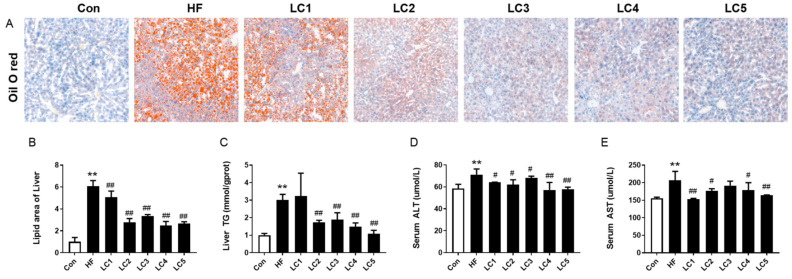
Effect of L-carnitine on hepatic lipid accumulation and liver function in NAFLD mice. (**A**) Results of the Oil O red-stained liver in different groups (scale bar, 200 μm); (**B**) quantified lipid area (**A**) (calculated using ImageJ), *n* = 3 each group for (**A**,**B**); (**C**) hepatic TG levels; (**D**,**E**) serum levels of ALT and AST, *n* = 10 each group for (**C**–**E**); ** *p*< 0.01 to Con; ^#^ *p* < 0.05, ^##^ *p* < 0.01 to HF.

As shown in Figure 2A,B, the mice in the HF group had more extensive red-dyed lipid droplets in their livers (*p* < 0.01). L-carnitine supplementation dramatically reduced the areas of lipid droplets in a dose–effect relationship. Of note, the enhanced hepatic TG levels in the HFD-fed mice were remarkably reduced in the LC2-5 groups (*p* < 0.05) (Figure 2C). The serum levels of ALT and AST are well-known biochemical markers of liver function. As presented in Figure 2D,E, the mice in the LC1-5 groups had lower serum ALT levels than those in the HF group (*p* < 0.05). L-carnitine (0.2%, 0.5%, 2% and 4%) supplementation significantly decreased the elevated serum AST levels (*p* < 0.05) compared to the HF group. Collectively, these data prove that L-carnitine treatment effectively ameliorated hepatic lipid deposition and dysfunction induced by HFD.

### 3.3. L-Carnitine Supplementation Attenuated Hepatic Inflammation

**Figure 3:**
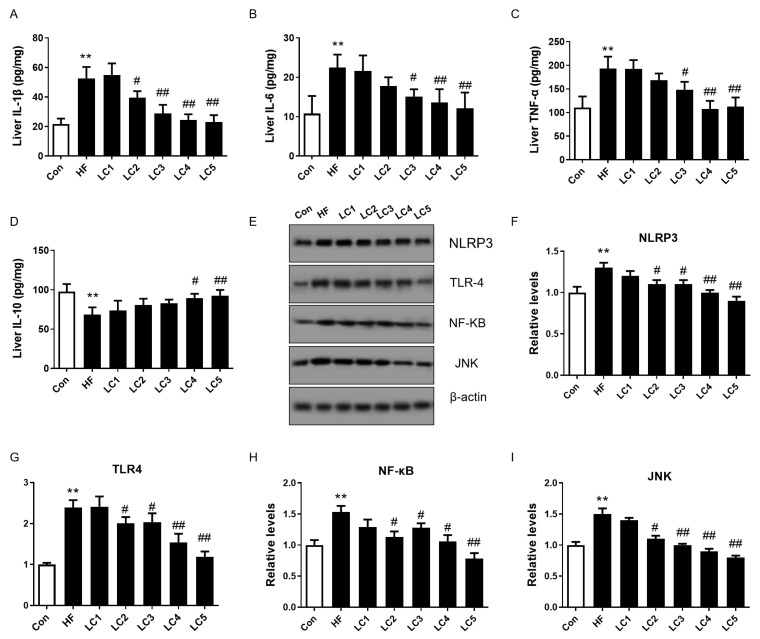
Effect of L-carnitine on hepatic inflammation in HFD-induced NAFLD mice. (**A**–**D**) Concentrations of IL-1β, IL-6, TNF-α and IL-10 in liver tissue (picograms per milligram of protein), *n* = 5 each group; (**E**) Western blot bands of proteins related to inflammatory-related pathways in liver; (**F**–**I**) relative protein levels of NLRP3, TLR4, NF-κB, JNK, *n* = 3 each group for (**E**–**I**); ** *p* < 0.01 to Con; ^#^ *p* < 0.05, ^##^ *p* < 0.01 to HF.

As displayed in Figure 3A–D, the mice in the HF group showed a marked increase in hepatic concentrations of IL-6, IL-1β and TNF-α and a decline in IL-10 compared with the Con group (*p* < 0.01). L-carnitine (1%, 2%, 4%) supplementation significantly reduced the hepatic levels of IL-6, IL-1β and TNF-α in the HFD-fed mice (*p* < 0.05). The LC2 group also displayed a greater decline in IL-1β than the HF group (*p* < 0.05). The hepatic levels of IL-10 were enhanced in the LC4 and LC5 groups compared to the HF group (*p* < 0.05). We also measured the relative protein expressions of genes related to inflammatory-related pathways in the liver (Figure 3E–I), and the protein expressions of NLRP3, TLR-4, NF-κB and JNK in the liver were obviously higher in the HF group than in the Con group (*p* < 0.01). Except for the 0.2% L-carnitine-treated mice, the mice in the other LC groups presented notably lower relative protein expressions of these inflammation-related genes than the mice in the HF group (*p* < 0.05).

### 3.4. Lipidomics Analysis of Hepatic Lipid Profiles following L-Carnitine Supplementation

**Figure 4:**
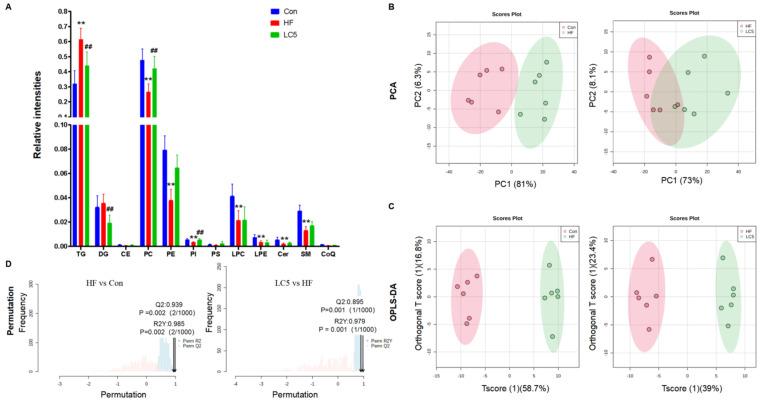
Lipidomics analysis of hepatic lipid profiles in the experimental mice. (**A**) Bar diagram representing the relative intensities of lipid species in different groups; (**B**) PCA scores plots; (**C**) OPLS-DA of HF versus Con and LC5 versus HF groups, respectively; (**D**) permutation test of the OPLS-DA models; *n* = 6 each group; HF vs. Con: the intercepts of R2 = 0.985, Q2 = 0.939; LC5 vs. HF: R2 = 0.979, Q2 = 0.895. ** *p* < 0.01 to Con; ## *p* < 0.01 to HF.

To identify the changes in lipid species among the Con, HF and LC5 groups, a lipidomics approach was utilized. A total of 12 classes and 145 lipid species were detected in the liver (Figure 4A and Supplementary Table S1). As expected, TGs and PC comprised the bulk of the total lipid. A serious disturbance in the lipid profiles was found in the mice fed an HFD, characterized by a higher relative abundance of TGs and lower levels of PC, phosphatidylethanolamine (PE), phosphatidylinositol (PI), lyso-phosphatidylcholine (LPC), lyso-phosphatidylethanolamine (LPE), ceramide (Cer) and sphingomyelin (SM) than the Con group (*p* < 0.01 for all). However, the relative levels of the PC and PI classes were significantly raised, and those of the TG and DG classes were significantly reduced by the 4% LC administration (*p* < 0.05, Figure 4A). This finding suggests that the HFD-induced perturbation of lipid metabolism was partially attenuated by L-carnitine. No change was observed in cholesteric esters (CEs), phosphatidylserine (PS) or coenzyme Q (CoQ) among the groups.

The results of a PCA analysis are shown in Figure 4B. A marked separation was noticed between the Con and HF groups, while the HF and LC5 groups partially overlapped. To obtain a higher grade of separation and a better understanding of the lipid alterations among the groups, we further conducted an OPLS-DA analysis. As shown in Figure 4C, a marked separation between the HF and Con or LC5 groups was found in the OPLS-DA models. Furthermore, we performed a 1000-times permutation test to evaluate the OPLS-DA models, and two multivariate models were obtained. The validation parameters of fitness and predictability (R2Y = 0.985 and Q2 = 0.939) in HF versus Con, as well as (R2Y = 0.979 and Q2 = 0.835, respectively) in LC5 versus HF, were all higher than 0.5, suggesting a vigorous fit and prediction of the models (Figure 4D).

Furthermore, potential differential lipid biomarkers were selected according to a combination of VIP ≥ 1 and *p* < 0.05. There were 88 differential lipid species that could distinguish Con from HF and 67 that could distinguish HF from LC5 (Supplementary Tables S2 and S3). As shown in Table 1, about 47 overlapped lipid species were selected in triple groups, namely, 29 TGs, 2 DGs, 7 PCs, 5 PEs, 2 SMs, 1 Cer and 1 PI. Among the 29 TGs species acquired, 20 TGs with a higher number of double bonds were significantly increased, and 9 TGs with lower numbers of double bonds were decreased in HF versus Con. In addition, the contents of two DGs that contained oleates and were monounsaturated (DG(18:1_18:1) and DG(16:0_18:1)) and PC(40:5) were markedly increased, and six PCs (PC(17:1_20:5), PC(34:2), PC(35:2), PC(35:4), PC(38:6) and PC(40:7)), five PEs (PE(16:0_18:2), PE(16:0_20:4), PE(16:0_22:6), PE(18:1_20:4) and PE(18:1_22:6)), PI(18:0_20:4), two SMs (SM(d18:1_24:1) and SM(d34:1)) and Cer(d18:1_16:0) were decreased in the HF versus Con groups. Interestingly, LC5 supplementation significantly reversed the relative levels of all these lipids, except for PC(40:5).

### 3.5. Correlation Analysis

**Figure 5:**
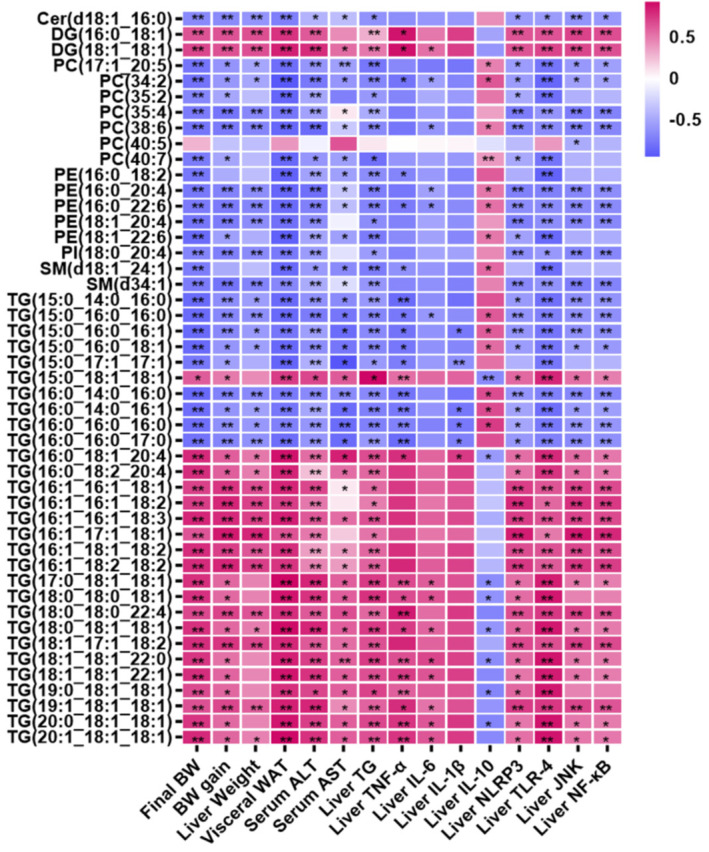
Spearman’s correlations between significantly differential lipid biomarkers with parameters related to NAFLD in the Con, HF and LC5 groups. * *p* < 0.05, ** *p* < 0.01.

To further explore the L-carnitine-modulated lipid species in NAFLD, we analyzed the correlations between the differential lipid species with parameters related to NAFLD using Spearman’s correlation analysis. As shown in Figure 5, 20 TGs ((15:0_18:1_18:1), (16:0_18:1_20:4), (16:0_18:2_20:4), (16:1_16:1_18:1), (16:1_16:1_18:2), (16:1_16:1_18:3), (16:1_17:1_18:1), (16:1_18:1_18:2), (16:1_18:2_18:2), (17:0_18:1_18:1), (18:0_18:0_18:1), (18:0_18:0_22:4), (18:0_18:1_18:1), (18:1_17:1_18:2), (18:1_18:1_22:0), (18:1_18:1_22:1), (19:0_18:1_18:1), (19:1_18:1_18:1), (20:0_18:1_18:1) and (20:1_18:1_18:1)) and 2 DGs ((16:0_18:1) and (18:1_18:1)) exhibited positive correlations with most of the NAFLD parameters and were negatively correlated with liver IL-10 (*p* < 0.05). In contrast, negative associations between nine TGs with saturated and monounsaturated double bonds ((15:0_14:0_16:0), (15:0_16:0_16:0), (15:0_16:0_16:1), (15:0_16:0_18:1), (15:0_17:1_17:1), (16:0_14:0_16:0), (16:0_14:0_16:1), (16:0_16:0_16:0) and (16:0_16:0_17:0)), six PCs ((17:1_20:5), (34:2), (35:2), (35:4), (38:6) and (40:7)), five PEs ((16:0_18:2), (16:0_20:4), (16:0_22:6), (18:1_20:4) and (18:1_22:6)), two SMs ((d18:1_24:1) and (d34:1)), Cer(d18:1_16:0) and PI(18:0_20:4) with the majority of NAFLD-related parameters were observed. In addition, PC(40:5) was negatively correlated with JNK in the livers.

### 3.6. Pathway Analysis

**Figure 6:**
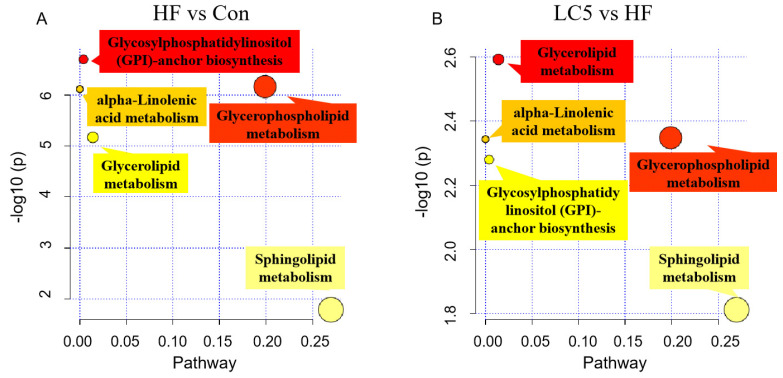
Pathway analysis based on differential lipid species in HF vs. Con (**A**) and LC5 vs. HF (**B**). The node colors (yellow and red) are based on *p*-values (*Y*-axis, yellow indicates higher *p* values; red indicates lower *p* values), and the node size determines impact values of pathway (*X*-axis, the larger the size, the higher the impact score).

To have a better understanding of the roles of the identified lipid metabolites in the process of L-carnitine regulating NAFLD, a pathway analysis was conducted. As shown in Figure 6, the mice being fed an HFD clearly resulted in the activation of the pathways of glycerolipid metabolism and inhibited the pathways of Glycosylphosphatidylinositol (GPI)-anchor biosynthesis, glycerophospholipid metabolism, alpha-linolenic acid metabolism and sphingolipid metabolism in the liver as compared to the Con group. However, LC supplementation inhibited the glycerolipid metabolism pathway and activated the pathways of alpha-linolenic acid metabolism, glycerophospholipid metabolism, sphingolipid metabolism and GPI-anchor biosynthesis.

## 4. Discussion

L-carnitine is an essential nutrient that is beneficial for lipid metabolism. In the current study, we proved that L-carnitine (0.5–4%) dietary supplementation could effectively lower body weight, liver weight, the weight of visceral WAT, hepatic lipid accumulation and the levels of AST and ALT in serum. These results suggest that L-carnitine can significantly improve HFD-induced NAFLD, and they are consistent with those of previous studies [27,28]. Mechanistic studies showed that L-carnitine reduced HFD-induced chronic inflammation in the liver. A lipidomics analysis identified 47 lipid species able to distinguish the experimental groups. A pathway analysis revealed that L-carnitine activated the pathways of glycerophospholipid metabolism, alpha-linolenic acid metabolism, sphingolipid metabolism and GPI-anchor biosynthesis in the liver of the NAFLD mice and inhibited the pathway of glycerolipid metabolism.

NAFLD is a complex and multifactorial disease characterized by low-grade chronic inflammation [29]. In this context, the inflammatory microenvironment contributes to the perpetuation of liver damage through the action of several pro-inflammatory factors, such as IL-1β, IL-6 and TNF-α, that in turn exacerbate liver inflammation [30]. These pro-inflammatory cytokines activate various inflammatory pathways that interfere with the development of NAFLD [31], such as the inhibitor kappa B kinase beta/nuclear factor kappa B (IKK/NF-kB) pathway and the c-Jun *N*-terminal kinase/activator protein 1 (JNK/AP1) pathway [32,33]. IL-10 is an anti-inflammatory cytokine that could inhibit the TLRs-NF-kB signaling pathway [34]. Recently, the NLRP3 inflammasome, an intracellular multi-protein complex, was recognized to be closely associated with hepatic inflammation and the progression of NAFLD [35,36]. During NAFLD, multiple danger signaling molecules, such as LPS and ROS, connect with the TLR4 receptor to activate the NF-κB and JNK inflammatory pathways, and they further trigger the activation of the NLRP3 inflammasome pathway. The activation of the NLRP3 inflammasome leads to the maturation of the pro-inflammatory cytokines IL-1β and IL-18, which aggravate hepatic inflammation and promote pyroptosis [37]. Herein, we found that L-carnitine supplementation significantly reduced the hepatic levels of IL-6, IL-1β and TNF-α and enhanced IL-10 compared to those in the NAFLD mice. The potential mechanisms might be related to the inhibition of the TLR-4/NF-κB/JNK inflammatory pathway and the activation of the NLRP3 inflammasome in the liver. In mice with NAFLD induced by a methionine- and choline-deficient diet, L-carnitine supplementation attenuated NAFLD, ameliorated systemic inflammation and suppressed the expression of NF-kB in the liver [10]. In rats with liver damage induced by alcohol [38] or acetate [39], L-carnitine alleviated hepatic inflammation. These findings are consistent with our results. Hence, the results suggest that L-carnitine might ameliorate NAFLD by inhibiting hepatic inflammation.

To further explore the potential mechanisms underlying the amelioration of NAFLD by L-carnitine, we conducted lipidomics analyses. In recent years, lipidomics analyses have been used to explore the pathogenesis of various chronic diseases, such as NAFLD [20]. Alterations in multiple hepatic lipid species, such as glycerophospholipids (PC, PE, LPC and LPE), glycerolipids (TG and DG) and sphingolipids (Cer and SM), have been shown to correlate with NAFLD [40]. In this paper, we comprehensively examined the lipid profiles in the livers of the experimental mice by using the lipidomics method and identified 47 lipid species, namely, 29 TGs, 2 DGs, 7 PCs, 5 PEs, 2 SMs, 1 Cer and 1 PI, to distinguish the L-carnitine-treated mice from the NAFLD mice.

Glycerides contain a glycerol skeleton and fatty acids. Based on the number of fatty acids, glycerides are classified into monoglycerides, DGs and TGs. Among which, TG is an important glyceride that is involved in the process of NAFLD [41]. TG accumulation in lipid droplets of the liver is the first step of NAFLD development [42,43]. A lipidomics analysis of the human liver showed that NAFLD is related to an increase in major species of TG [44]. Similar results were observed in HFD-fed NAFLD mice [19,22]. In our study, most TG species were increased in the liver of the HFD-induced NAFLD mice. Specially, 20 TGs with a higher number of double bonds were increased, and 9 TGs with less double bonds were markedly decreased following HFD feeding. These findings are in line with the reports of Feng et al. [21], who found that, in Western diet-induced NAFLD mice, 35 TG species were increased, while the TG species with lower numbers of double bonds (46:0, 48:0, 48:1, 50:1 and 58:2) were decreased. These results indicate that different TG species might play distinct roles during the onset of NAFLD [45,46]. Although saturated fatty acids are known to be metabolically harmful and fatty acids with a high degree of unsaturation are beneficial for health, TG serving as a storage lipid is metabolically inert. Thus, the fatty acids stored in TG are not the primary determinant of lipotoxicity [47]. In this study, LC5 supplementation significantly decreased the levels of the 20 TGs with a higher number of double bonds and increased the 9 TGs with less double bonds, indicating that the ameliorative role of L-carnitine in NAFLD was closely related to these TC species.

A remarkable elevation in DG species is another hallmark of NAFLD [48]. Increased oleate-containing and monounsaturated fatty acid DGs were observed in the liver of humans with obesity and positively associated with insulin resistance [49]. DGs are recognized as mediators of insulin resistance, and they accumulate in the liver during NAFLD [50]. Notably, the present study revealed an increase in two DGs that contained oleates and were monounsaturated (DG (18:1_18:1) and DG (16:0_18:1)) in the liver of the NAFLD mice. Moreover, the results of the correlation analysis further suggest that the two DGs were positively related with the outcomes of NAFLD. The findings support the notion that DGs might promote the progression of NAFLD. Moreover, 4% L-carnitine significantly reduced the aforementioned DG species. The pathway analysis revealed that the activated glycerolipid metabolism was inhibited by L-carnitine.

Glycerophospholipids are important structural components of cell membranes and participate in various cell signaling transduction pathways [51]. An imbalance in the metabolism of glycerophospholipids might disrupt the stability of the membrane and promote NAFLD [52]. PC and PE are the two major glycerophospholipids in mammals. PC plays important roles in exporting neutral lipids (e.g., TGs) out of the liver by assembling very-low-density lipoprotein (VLDL). Decreased PC levels in the liver will inhibit the synthesis of VLDL and increase the risk of NAFLD [21]. In mice and humans, approximately 70% of hepatic PC is produced via the cytidine diphosphate (CDP)-choline pathway and 30% via the phosphatidylethanolamine *N*-methyltransferase (PEMT) pathway, during which PE is converted into PC via sequential methylation reactions. PE also regulates membrane fusion, and it regulates NAFLD. Decreased hepatic levels of both PC and PE were reported in rats challenged with CCl4 [53]. These reports are in line with our findings that the relative abundance of both PC and PE were declined in the NAFLD mice. Moreover, increasing evidence suggests that the homeostasis of glycerophospholipids is linked to the metabolism of other lipid classes, such as glycerolipids. When the synthesis of PC or PE is suppressed, more DG will be converted into TG [52]. Herein, numerous glycerophospholipid species were converted in the HFD-induced mice and were reversed by L-carnitine. Briefly, we found that several GPs were decreased in the NAFLD mice, including six PCs (PC(17:1_20:5), PC(34:2), PC(35:2), PC(35:4), PC(38:6) and PC(40:7)), five PEs (PE(16:0_18:2), PE(16:0_20:4), PE(16:0_22:6), PE(18:1_20:4) and PE(18:1_22:6)) and PI(18:0_20:4). However, almost all these lipids were upregulated after L-carnitine intervention. In addition, these glycerophospholipids exhibited clear negative correlations with NAFLD-related indices. The results provide further support for the notion that glycerophospholipids play critical roles in NAFLD [21,54]. Furthermore, the pathway analysis also indicated that L-carnitine improved the liver pathway of glycerophospholipid metabolism in the NAFLD mice.

Sphingolipid metabolism is involved in NAFLD development in several ways [55,56]. The liver is a primary production tissue for sphingolipids, especially Cer and SM [57]. Cer is involved in various bioactivities, such as cell differentiation, apoptosis and autophagy, while SM regulates cell proliferation and survival [58]. The dysregulation of sphingolipid metabolism drove the progression of NAFLD [59]. In this study, both total Cer and SM were reduced in the liver of the NAFLD mice. We also observed that SM(d18:1_24:1), SM(d34:1) and Cer(d18:1_16:0) were markedly decreased in the HF versus Con groups. The correlation analysis showed that they were negatively correlated with NAFLD. Similar observations were reported by Sun et al. that some SM species, such as SM(d18:1/16:0), were dramatically reduced in NAFLD mice [59]. L-carnitine supplementation significantly increased the hepatic levels of the three sphingolipids. The pathway analysis also indicated that L-carnitine might ameliorate NAFLD by improving the sphingolipid metabolism pathway in the liver.

Moreover, several reports revealed that the deficiency of alpha-Linolenic acid (ALA) in serum and the liver is a common feature of patients with non-alcoholic [60] and alcoholic liver disease [61]. ALA belongs to long-chain omega-3 polyunsaturated fatty acids and is beneficial for preventing NAFLD by ameliorating inflammation and improving insulin sensitivity [62,63]. Herein, we found that HFD feeding clearly inhibited the alpha-linolenic acid metabolism in mice, and this is consistent with previous reports [22]. Conversely, L-carnitine ameliorated this pathway. As is known, ALA cannot be naturally synthesized in vivo and is an essential dietary nutrient for all mammals. The beneficial effects of L-carnitine on ALA metabolism would be an interesting target for explaining its protective roles in NAFLD.

Meanwhile, we found that the Glycosylphosphatidylinositol (GPI)-anchor biosynthesis pathway was blocked in the NAFLD mice. In humans, over 150 kinds of proteins are anchored to the cell membrane through a GPI form, such as various adhesion molecules, receptors and complement regulators [64]. The enzyme GPI-specific phospholipase D (GPI-PLD) can hydrolyze the glycan-phosphatidylinositol linkages of GPI anchors [65] and is abundant in the blood circulation of mammals [66,67]. Patients with NAFLD have elevated GPI-PLD in their blood and liver [68]. GPI-PLD overexpression is also related to an enhanced expression of genes controlling de novo lipogenesis [68]. These pieces of evidence suggest that the GPI anchor plays a protective role in NAFLD [68]. In this study, L-carnitine treatment activated this pathway.

## 5. Conclusions

**Figure 7:**
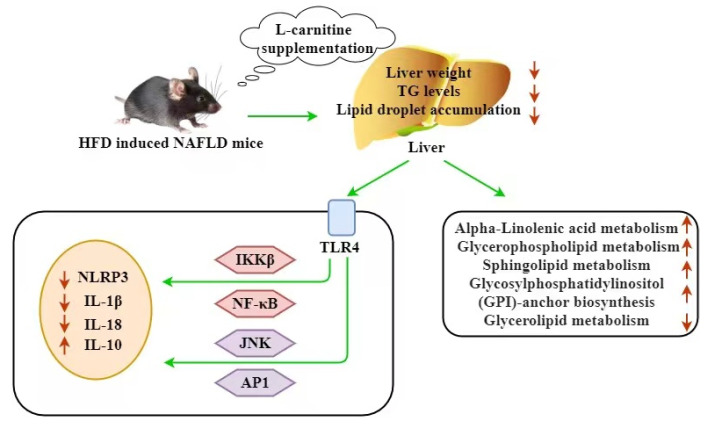
L-carnitine alleviated NAFLD by modulating hepatic inflammation and lipidomics in HFD-fed mice.

Our study provides further evidence that L-carnitine treatment can effectively alleviate NAFLD in HFD-fed mice. The beneficial effects might be attributed to the modulation of hepatic inflammation. Importantly, we provide novel insights into the mechanisms of dietary L-carnitine in NAFLD using a lipidomics approach. In particular, the lipidomics results show that L-carnitine modulated several lipid biomarkers and significantly improved the liver pathways of glycerophospholipid metabolism, sphingolipid metabolism, alpha-linolenic acid metabolism and GPI-anchor biosynthesis and inhibited the pathway of glycerolipid metabolism in the HFD-induced NAFLD mice (Figure 7). Further studies are warranted to deeply explore the roles of the identified lipid biomarkers and pathways in the protective roles of L-carnitine in NAFLD.

## Figures and Tables

**Table 1:** Significantly changed hepatic lipid species among groups based on the criteria of VIP ≥ 1 and *p* < 0.05.

Lipid Species	HF vs. Con			LC5 vs. HF		
	^a^ VIP	^b^ FC	^c^ Trend	VIP	FC	Trend
Cer(d18:1_16:0)	1.03	0.52	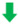	1.03	2.17	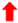
DG(16:0_18:1)	1.06	2.06	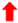	1.24	0.52	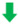
DG(18:1_18:1)	1.14	2.44	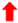	1.29	0.48	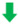
PC(17:1_20:5)	1.18	0.48	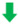	1.39	1.61	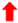
PC(34:2)	1.25	0.30	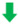	1.11	1.99	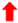
PC(35:2)	1.10	0.36	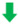	1.05	1.56	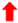
PC(35:4)	1.01	0.63	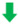	1.37	1.62	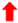
PC(38:6)	1.20	0.54	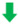	1.45	1.78	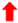
PC(40:5)	1.02	2.01	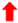	1.21	2.04	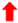
PC(40:7)	1.18	0.48	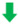	1.14	1.46	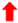
PE(16:0_18:2)	1.27	0.27	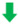	1.17	1.76	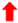
PE(16:0_20:4)	1.10	0.53	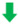	1.34	1.80	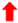
PE(16:0_22:6)	1.22	0.53	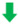	1.44	1.83	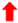
PE(18:1_20:4)	1.05	0.57	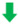	1.30	1.68	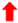
PE(18:1_22:6)	1.22	0.47	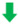	1.28	1.51	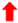
PI(18:0_20:4)	1.11	0.60	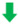	1.32	1.67	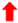
SM(d18:1_24:1)	1.26	0.35	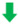	1.11	1.52	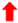
SM(d34:1)	1.14	0.62	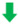	1.26	2.05	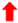
TG(15:0_14:0_16:0)	1.16	0.16	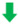	1.25	4.17	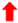
TG(15:0_16:0_16:0)	1.23	0.17	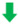	1.39	4.59	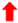
TG(15:0_16:0_16:1)	1.17	0.24	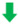	1.29	3.20	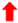
TG(15:0_16:0_18:1)	1.24	0.38	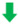	1.23	2.02	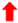
TG(15:0_17:1_17:1)	1.26	0.35	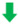	1.15	1.99	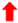
TG(15:0_18:1_18:1)	1.04	1.44	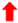	1.12	0.76	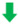
TG(16:0_14:0_16:0)	1.27	0.14	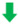	1.22	5.36	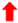
TG(16:0_14:0_16:1)	1.14	0.34	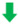	1.10	2.22	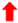
TG(16:0_16:0_16:0)	1.20	0.18	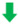	1.30	4.33	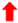
TG(16:0_16:0_17:0)	1.20	0.26	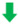	1.32	3.24	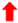
TG(16:0_18:1_20:4)	1.18	2.72	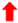	1.24	0.57	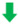
TG(16:0_18:2_20:4)	1.05	3.91	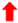	1.20	0.42	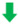
TG(16:1_16:1_18:1)	1.10	2.60	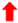	1.42	0.39	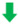
TG(16:1_16:1_18:2)	1.04	3.22	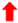	1.40	0.19	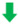
TG(16:1_16:1_18:3)	1.10	5.35	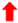	1.35	0.19	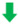
TG(16:1_17:1_18:1)	1.14	1.52	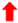	1.46	0.57	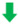
TG(16:1_18:1_18:2)	1.08	2.39	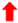	1.34	0.47	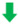
TG(16:1_18:2_18:2)	1.04	3.25	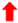	1.37	0.24	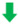
TG(17:0_18:1_18:1)	1.20	2.38	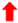	1.31	0.60	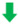
TG(18:0_18:0_18:1)	1.20	5.22	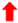	1.35	0.45	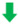
TG(18:0_18:0_22:4)	1.27	2.36	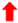	1.13	0.60	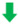
TG(18:0_18:1_18:1)	1.23	4.77	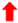	1.28	0.57	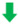
TG(18:1_17:1_18:2)	1.17	2.08	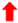	1.43	0.50	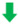
TG(18:1_18:1_22:0)	1.18	2.28	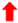	1.31	0.66	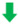
TG(18:1_18:1_22:1)	1.21	3.31	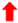	1.38	0.50	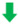
TG(19:0_18:1_18:1)	1.13	1.93	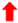	1.29	0.64	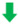
TG(19:1_18:1_18:1)	1.08	1.69	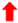	1.32	0.63	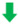
TG(20:0_18:1_18:1)	1.21	5.58	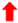	1.42	0.39	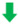
TG(20:1_18:1_18:1)	1.24	4.47	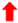	1.40	0.49	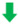

^a^ VIP was obtained from OPLS-DA; ^b^ FC was calculated based on mean ratios for HF vs. Con or LC5 vs. HF; ^c^ trend represents increase or decrease between the two groups.

## Data Availability

Not applicable.

## Supplementary Materials

The following supporting information can be downloaded at https://www.mdpi.com/article/10.3390/nu15061359/s1, Table S1: Peak areas of all the identified hepatic lipid species; Table S2: Deferential hepatic lipid species between HF vs. Con based on the criteria of VIP ≥ 1 and *p* < 0.05; Table S3: Deferential hepatic lipid species between LC5 vs. HF based on the criteria of VIP ≥ 1 and *p* < 0.05.
