# Supplementary material for: Liver Lipidomics Analysis Revealed the Novel Ameliorative Mechanisms of L-Carnitine on High-Fat Diet-Induced NAFLD Mice
Source: Nutrients. 2023 Mar 10;15(6):1359. doi: 10.3390/nu15061359 (PMC10053018; doi:10.3390/nu15061359)
Supplement: Supplementary file 1 [file nutrients-15-01359-s001.zip › nutrients-2223898-supplementary.pdf]

Supplemental Tables

Abbreviations of lipids

Cer, ceramide; CE, cholesteric esters; Co, coenzyme; DG, diacylglycerol; TG, triacylglycerol; PC, phosphatidylcholine; LPC, lyso-phosphatidylcholine; PE, phosphatidylethanolamine; LPE, lyso-phosphatidylethanolamine; PI, phosphatidylinositol; PS, phosphatidylserine; SM, sphingomyelin

Supplementary Table S1. Peak areas of all the identified hepatic lipid species.

Lipid species	Con（×10⁴）		HF（×10⁴）		LC5（×10⁴）	
	Mean	SD	Mean	SD	Mean	SD
Cer(d18:1_16:0)	5.08	±1.33	2.64	±0.79	5.71	±2.62
Cer(d18:1_22:0)	15.41	±6.03	8.39	±2.52	7.80	±1.26
Cer(d18:1_24:0)	14.30	±5.87	3.46	±1.17	4.66	±1.59
Cer(d18:1_24:1)	20.32	±5.87	5.71	±1.87	8.68	±3.25
CE(18:1)	4.95	±2.22	5.92	±2.63	7.36	±4.53
CE(18:2)	5.91	±3.26	0.13	±0.16	1.28	±0.58
Co(Q9)	15.14	±1.87	7.08	±1.52	8.28	±1.95
DG(16:0_18:1)	25.76	±10.51	53.08	±10.72	27.37	±11.69
DG(16:0_18:2)	64.51	±25.68	60.29	±10.84	29.60	±7.57
DG(16:0_22:6)	4.56	±1.50	4.11	±0.79	3.55	±0.48
DG(18:0_16:0)	14.97	±4.67	8.13	±2.26	6.73	±1.09
DG(18:0_20:4)	25.04	±5.28	18.04	±5.11	16.18	±8.69
DG(18:1_18:1)	35.94	±11.56	87.52	±18.70	42.22	±17.37
DG(18:1_18:2)	72.02	±25.56	73.08	±17.29	38.87	±10.43
DG(18:1_20:4)	9.64	±1.79	16.43	±3.61	8.94	±2.93
DG(18:1_22:6)	20.35	±8.71	12.96	±6.73	8.42	±2.82
DG(18:2_18:2)	31.22	±14.27	17.92	±6.22	8.18	±1.65
DG(18:2_22:6)	19.98	±9.91	4.40	±2.62	4.16	±0.83
LPC(15:0)	48.68	±12.42	27.52	±10.77	24.53	±13.96
LPC(16:0)	171.90	±36.52	80.45	±27.62	75.24	±37.17
LPC(18:0)	57.51	±11.90	41.85	±13.28	44.71	±19.28
LPC(18:1)	11.20	±3.37	9.65	±2.91	12.32	±6.39
LPC(18:2)	61.53	±18.23	18.01	±9.38	19.38	±9.54
LPC(20:4)	30.54	±8.79	21.89	±8.28	23.28	±13.32
LPC(22:6)	32.90	±12.12	15.77	±7.04	18.22	±11.84
LPE(16:0)	29.91	±9.03	12.29	±5.33	11.95	±6.77
LPE(18:1)	16.66	±4.51	7.09	±3.01	5.03	±3.06
LPE(20:4)	10.42	±3.33	6.33	±2.39	6.62	±3.98
LPE(22:6)	16.08	±6.61	6.26	±2.78	6.93	±4.98
PC(16:0_18:1)	293.14	±34.31	274.16	±84.56	383.77	±86.41
PC(17:1_20:5)	121.04	±22.65	57.79	±15.21	92.94	±9.10
PC(18:0_18:2)	285.02	±56.06	140.05	±39.93	191.02	±53.16
PC(18:0_20:3)	38.16	±6.38	40.02	±12.16	26.28	±3.55
PC(32:0)	65.41	±7.26	24.74	±6.63	32.75	±7.82
PC(32:1)	32.96	±5.77	19.29	±4.52	23.68	±6.21
PC(34:0)	13.08	±2.81	7.89	±2.55	7.41	±2.17
PC(34:2)	1303.76	±183.18	393.99	±111.17	785.62	±279.87

PC(34:3)	82.53	±9.81	16.05	±4.58	18.55	±5.64
PC(35:2)	10.97	±3.25	3.97	±1.31	6.21	±1.43
PC(35:4)	273.73	±55.28	173.24	±38.59	279.83	±36.03
PC(36:1)	43.85	±7.28	44.75	±12.31	42.67	±8.66
PC(36:3)	66.26	±9.98	99.18	±24.43	64.71	±30.50
PC(36:4)	709.28	±176.05	494.83	±91.04	828.04	±133.08
PC(36:5)	58.58	±11.56	12.12	±5.72	19.94	±11.94
PC(37:5)	2.34	±0.27	2.58	±0.92	6.37	±1.30
PC(38:4)	221.19	±53.78	222.87	±30.23	332.68	±72.19
PC(38:5)	161.46	±36.75	103.94	±17.20	162.18	±34.90
PC(38:6)	707.22	±102.20	379.80	±66.33	676.11	±89.31
PC(40:5)	2.00	±0.34	4.02	±1.37	8.21	±2.69
PC(40:6))	97.33	±19.12	67.82	±11.48	106.79	±24.32
PC(40:7)	132.47	±23.72	62.99	±13.32	91.94	±17.97
PC(40:8)	32.44	±7.58	11.52	±3.85	15.08	±6.14
PC(42:10)	6.32	±1.80	3.39	±1.62	5.13	±3.74
PE(16:0_18:2)	68.62	±7.02	18.83	±6.35	33.08	±8.23
PE(16:0_20:4)	106.77	±21.05	56.56	±12.75	102.02	±19.53
PE(16:0_22:6)	290.29	±35.71	153.16	±34.86	280.07	±39.08
PE(16:0p_22:6)	7.82	±0.87	2.44	±0.68	5.24	±1.41
PE(18:0_18:2)	51.23	±9.53	18.71	±6.22	24.84	±4.90
PE(18:0p_20:4)	11.79	±2.42	8.52	±2.02	13.47	±3.39
PE(18:1_18:2)	55.67	±10.08	14.43	±6.16	15.95	±4.95
PE(18:1_20:4)	121.91	±26.99	69.60	±14.22	116.70	±22.04
PE(18:1_22:6)	81.15	±11.67	37.76	±9.84	57.01	±7.54
PI(18:0_20:4)	53.45	±9.17	32.14	±5.89	53.80	±9.49
PS(18:0_20:4)	14.08	±3.62	8.88	±4.42	22.05	±16.42
SM(d18:1_22:0)	94.11	±18.39	56.97	±14.45	56.89	±8.15
SM(d18:1_24:1)	73.18	±9.68	25.72	±5.91	39.17	±9.94
SM(d34:1)	30.25	±2.40	18.62	±4.79	38.15	±11.37
SM(d38:1)	9.35	±1.22	8.59	±1.99	9.56	±0.96
SM(d41:1)	23.11	±6.02	6.89	±1.73	8.38	±1.82
SM(d42:1)	61.90	±12.74	14.11	±5.08	18.71	±6.04
TG(15:0_14:0_16:0)	29.89	±9.78	4.79	±1.70	19.97	±9.70
TG(15:0_16:0_16:0)	54.64	±11.89	9.50	±6.28	43.59	±14.29
TG(15:0_16:0_16:1)	63.36	±16.39	15.41	±6.34	49.31	±19.24
TG(15:0_16:0_18:1)	68.96	±9.06	26.31	±5.33	53.02	±16.57
TG(15:0_16:1_16:1)	30.90	±4.66	11.23	±2.48	18.58	±4.89
TG(15:0_17:1_17:1)	64.89	±6.22	22.63	±4.67	45.03	±15.80
TG(15:0_18:1_18:1)	45.89	±3.50	65.88	±12.48	50.28	±5.73
TG(15:0_18:2_18:2)	5.64	±2.01	9.76	±2.72	3.90	±0.58
TG(16:0_14:0_16:0)	60.99	±8.48	8.70	±4.06	46.65	±25.13
TG(16:0_14:0_16:1)	60.82	±13.95	20.87	±5.58	46.37	±20.30
TG(16:0_16:0_16:0)	80.20	±20.15	14.34	±8.30	62.13	±25.86
TG(16:0_16:0_16:1)	118.31	±28.78	39.79	±11.53	81.19	±40.04
TG(16:0_16:0_17:0)	31.95	±5.87	8.41	±5.39	27.24	±8.08
TG(16:0_16:0_18:1)	126.34	±59.55	134.19	±29.53	159.42	±46.44

TG(16:0_16:1_16:1)	82.49	±25.74	64.85	±20.71	66.47	±26.46
TG(16:0_16:1_18:1)	162.11	±87.97	266.94	±62.10	191.54	±45.64
TG(16:0_17:0_18:1)	29.10	±3.45	21.62	±4.22	28.01	±6.06
TG(16:0_18:1_18:1)	215.90	±102.31	838.31	±87.22	637.01	±205.30
TG(16:0_18:1_18:2)	316.02	±173.02	832.71	±71.06	626.22	±229.20
TG(16:0_18:1_20:3)	140.87	±46.39	251.71	±25.75	203.81	±55.17
TG(16:0_18:1_20:4)	111.76	±42.36	303.94	±52.63	172.78	±64.28
TG(16:0_18:1_21:0)	6.60	±0.65	7.32	±1.13	7.90	±1.19
TG(16:0_18:1_22:0)	8.48	±0.95	16.59	±4.21	12.05	±2.68
TG(16:0_18:1_22:6)	55.45	±44.31	207.44	±64.40	120.56	±59.85
TG(16:0_18:1_24:0)	5.03	±0.23	5.11	±0.47	6.39	±1.05
TG(16:0_18:2_20:4)	47.29	±49.53	184.78	±62.52	76.89	±37.81
TG(16:0_18:2_22:6)	50.94	±42.72	103.14	±50.95	41.90	±23.56
TG(16:0_18:3_22:6)	4.47	±3.35	8.91	±6.00	2.12	±1.34
TG(16:0_20:3_22:6)	23.34	±14.95	28.84	±12.47	14.66	±6.32
TG(16:0_20:4_22:6)	1.48	±0.91	7.07	±6.44	3.32	±2.12
TG(16:0_22:1_22:6)	1.21	±0.42	2.41	±0.27	2.79	±0.96
TG(16:0_22:4_22:6)	0.85	±0.34	3.28	±2.41	2.67	±0.96
TG(16:0_22:5_22:6)	1.85	±1.18	4.28	±2.82	2.34	±0.96
TG(16:0_22:6_22:6)	0.69	±0.54	2.18	±1.65	0.95	±0.60
TG(16:1_14:0_16:1)	23.92	±2.56	12.34	±2.09	14.37	±4.29
TG(16:1_16:1_16:1)	20.63	±4.79	18.23	±6.59	12.22	±2.08
TG(16:1_16:1_17:1)	15.30	±1.34	7.69	±1.71	9.71	±2.09
TG(16:1_16:1_18:1)	88.71	±50.39	230.68	±60.72	89.54	±20.98
TG(16:1_16:1_18:2)	15.78	±11.28	50.82	±18.48	9.82	±2.26
TG(16:1_16:1_18:3)	0.81	±0.47	4.32	±1.69	0.81	±0.45
TG(16:1_17:1_18:1)	27.30	±2.64	41.59	±6.12	23.89	±2.72
TG(16:1_18:1_18:2)	222.16	±144.73	531.97	±87.22	248.66	±115.22
TG(16:1_18:1_23:0)	3.90	±0.34	6.10	±1.14	4.97	±0.63
TG(16:1_18:2_18:2)	41.87	±34.23	135.99	±45.34	32.27	±18.26
TG(16:1_18:2_18:3)	3.00	±2.73	13.62	±7.11	2.18	±1.23
TG(16:1_18:2_20:4)	13.10	±17.28	46.47	±22.96	8.45	±5.08
TG(17:0_18:1_18:1)	18.76	±1.97	44.61	±9.21	26.86	±5.41
TG(17:0_18:1_20:3)	4.57	±0.58	5.31	±0.84	4.10	±0.66
TG(18:0_16:0_16:0)	36.32	±10.44	24.26	±7.87	38.20	±15.29
TG(18:0_16:0_18:0)	18.87	±3.07	17.38	±5.08	19.30	±5.85
TG(18:0_16:0_18:1)	50.38	±27.56	141.84	±34.02	107.44	±31.09
TG(18:0_17:0_18:1)	10.81	±1.42	10.31	±1.64	10.01	±1.93
TG(18:0_18:0_18:0)	10.33	±1.86	9.25	±2.93	10.58	±2.98
TG(18:0_18:0_18:1)	15.10	±3.71	78.80	±22.94	35.41	±6.84
TG(18:0_18:0_22:4)	4.72	±0.63	11.12	±1.00	6.63	±3.11
TG(18:0_18:1_18:1)	50.05	±21.18	238.84	±55.12	135.78	±35.02
TG(18:0_18:1_20:3)	21.43	±3.59	42.50	±5.24	34.03	±6.74
TG(18:0_18:1_20:4)	27.70	±8.02	102.55	±16.01	75.49	±25.90
TG(18:0_18:1_22:5)	6.72	±2.06	21.87	±3.52	18.17	±5.40
TG(18:0_18:1_22:6)	10.25	±4.40	26.77	±4.28	23.42	±9.52
TG(18:1_17:1_18:2)	9.01	±2.58	18.78	±2.90	9.42	±2.40

TG(18:1_18:1_18:1)	112.18	±39.65	226.62	±19.91	208.59	±52.34
TG(18:1_18:1_20:4)	36.67	±16.90	135.30	±23.63	97.00	±40.19
TG(18:1_18:1_22:0)	5.64	±0.42	12.86	±2.67	8.55	±0.38
TG(18:1_18:1_22:1)	4.90	±0.71	16.21	±3.74	8.07	±2.02
TG(18:1_18:2_22:5)	19.77	±11.67	41.26	±11.95	30.92	±10.69
TG(18:2_17:1_18:2)	2.66	±1.46	5.66	±2.09	2.91	±0.83
TG(18:2_18:2_18:2)	65.77	±24.28	57.27	±20.04	18.27	±6.23
TG(18:2_18:2_22:6)	10.19	±7.53	5.11	±2.75	2.25	±0.91
TG(18:3_18:2_18:2)	8.50	±9.93	28.69	±11.21	7.26	±4.42
TG(19:0_18:1_18:1)	6.35	±0.86	12.22	±2.57	7.87	±1.08
TG(19:1_18:1_18:1)	6.43	±1.11	10.87	±2.22	6.85	±1.07
TG(20:0_18:1_18:1)	11.21	±1.99	62.61	±17.40	24.14	±4.53
TG(20:0_18:1_20:4)	5.01	±1.27	13.46	±1.52	12.28	±2.18
TG(20:1_18:1_18:1)	16.85	±5.66	75.26	±15.75	37.06	±10.26

Supplementary Table S2. Deferential hepatic lipid species between HF vs Con based on the criteria of VIP ≥ 1 and P<0.05.

	HF vs Con				HF vs Con		
**Lipid compounds**	**aVIP**	**bFC**	**cTrend**	**Lipid compounds**	**VIP**	**FC**	**Trend**
TG(16:0_22:1_22:6)	1.12	1.99	↑	TG(16:0_16:0_16:0)	1.20	0.18	↓
TG(16:0_18:1_22:0)	1.10	1.95	↑	TG(15:0_16:0_16:0)	1.23	0.17	↓
TG(18:0_18:1_20:3)	1.23	1.98	↑	TG(15:0_14:0_16:0)	1.16	0.16	↓
TG(19:0_18:1_18:1)	1.13	1.93	↑	TG(16:0_14:0_16:0)	1.27	0.14	↓
TG(16:0_18:1_20:3)	1.11	1.79	↑	TG(15:0_16:0_18:1)	1.24	0.38	↓
TG(19:1_18:1_18:1)	1.08	1.69	↑	TG(15:0_17:1_17:1)	1.26	0.35	↓
TG(16:1_18:1_23:0)	1.07	1.56	↑	TG(16:0_14:0_16:1)	1.14	0.34	↓
TG(16:1_17:1_18:1)	1.14	1.52	↑	TG(16:0_16:0_16:1)	1.15	0.34	↓
TG(15:0_18:1_18:1)	1.04	1.44	↑	TG(15:0_16:1_16:1)	1.20	0.36	↓
TG(18:0_18:1_22:6)	1.16	2.61	↑	TG(16:0_16:0_17:0)	1.20	0.26	↓
TG(16:0_18:1_22:6)	1.07	3.74	↑	TG(15:0_16:0_16:1)	1.17	0.24	↓
TG(18:0_18:1_22:5)	1.23	3.25	↑	DG(18:2_22:6)	1.06	0.22	↓
TG(18:0_18:0_22:4)	1.27	2.36	↑	PC(18:0_18:2)	1.10	0.49	↓
TG(18:1_18:1_22:1)	1.21	3.31	↑	PC(17:1_20:5)	1.18	0.48	↓
TG(18:1_18:1_22:0)	1.18	2.28	↑	PC(32:0)	1.25	0.38	↓
TG(20:0_18:1_18:1)	1.21	5.58	↑	PC(34:3)	1.25	0.19	↓
TG(20:1_18:1_18:1)	1.24	4.47	↑	PC(34:2)	1.25	0.30	↓
TG(20:0_18:1_20:4)	1.26	2.69	↑	PC(35:2)	1.10	0.36	↓
TG(18:0_18:0_18:1)	1.20	5.22	↑	PC(36:5)	1.22	0.21	↓
TG(18:0_18:1_18:1)	1.23	4.77	↑	PC(40:8)	1.18	0.36	↓
TG(16:1_16:1_18:3)	1.10	5.35	↑	PC(40:7)	1.18	0.48	↓
TG(16:0_18:2_20:4)	1.05	3.91	↑	PE(18:1_22:6)	1.22	0.47	↓
TG(16:0_18:1_18:1)	1.27	3.88	↑	PE(18:0_18:2)	1.20	0.37	↓
TG(18:0_18:1_20:4)	1.24	3.70	↑	PE(16:0p_22:6)	1.27	0.31	↓
TG(18:1_18:1_20:4)	1.21	3.69	↑	PE(16:0_18:2)	1.27	0.27	↓
TG(16:1_18:2_18:2)	1.04	3.25	↑	PE(18:1_18:2)	1.22	0.26	↓
TG(16:1_16:1_18:2)	1.04	3.22	↑	LPC(16:0)	1.14	0.47	↓
TG(18:0_16:0_18:1)	1.13	2.82	↑	LPC(18:2)	1.16	0.29	↓
TG(16:0_18:1_20:4)	1.18	2.72	↑	LPE(16:0)	1.09	0.41	↓
TG(16:0_18:1_18:2)	1.19	2.63	↑	LPE(18:1)	1.10	0.43	↓
TG(16:1_16:1_18:1)	1.10	2.60	↑	LPE(22:6)	1.03	0.39	↓
TG(16:1_18:1_18:2)	1.08	2.39	↑	SM(d18:1_24:1)	1.26	0.35	↓
TG(17:0_18:1_18:1)	1.20	2.38	↑	SM(d41:1)	1.19	0.30	↓
TG(18:1_17:1_18:2)	1.17	2.08	↑	SM(d42:1)	1.23	0.23	↓
TG(18:1_18:1_18:1)	1.18	2.02	↑	Cer(d18:1_24:1)	1.16	0.28	↓
DG(18:1_18:1)	1.14	2.44	↑	Cer(d18:1_24:0)	1.07	0.24	↓
DG(16:0_18:1)	1.06	2.06	↑	ChE(18:2)	1.10	0.02	↓
PC(40:5)	1.02	2.01	↑	Co(Q9)	1.24	0.47	↓
PC(38:6)	1.20	0.54	↓	PC(35:4)	1.01	0.63	↓
PE(16:0_20:4)	1.10	0.53	↓	PC(32:1)	1.02	0.59	↓
PE(16:0_22:6)	1.22	0.53	↓	PI(18:0_20:4)	1.11	0.60	↓
Cer(d18:1_16:0)	1.03	0.52	↓	PE(18:1_20:4)	1.05	0.57	↓
TG(16:1_14:0_16:1)	1.19	0.52	↓	SM(d34:1)	1.14	0.62	↓

TG(16:1_16:1_17:1)	1.18	0.50	↓	SM(d18:1_22:0)	1.03	0.61	↓

[a] VIP values obtained from OPLS-DA. [b] Fold change (FC) was calculated based on mean ratios for HF vs Con. [c] The icons of trend represent increase or reduced levels of lipid species between the two groups.

Supplementary Table S3. Deferential hepatic lipid species between LC5 vs HF based on the criteria of VIP ≥ 1 and P<0.05.

	LC5 vs HF				LC5 vs HF		
**Lipid compounds**	**[a]VIP**	**[b]FC**	**[c]Trend**	**Lipid compounds**	**VIP**	**FC**	**Trend**
TG(15:0_16:0_18:1)	1.23	2.02	↑	TG(16:1_18:2_18:3)	1.23	0.16	↓
TG(15:0_16:0_16:1)	1.29	3.20	↑	TG(16:1_18:2_20:4)	1.23	0.18	↓
TG(16:0_16:0_17:0)	1.32	3.24	↑	TG(16:1_16:1_18:3)	1.35	0.19	↓
TG(15:0_14:0_16:0)	1.25	4.17	↑	TG(16:1_16:1_18:2)	1.40	0.19	↓
TG(16:0_16:0_16:0)	1.30	4.33	↑	TG(16:1_18:2_18:2)	1.37	0.24	↓
TG(15:0_16:0_16:0)	1.39	4.59	↑	TG(16:0_18:3_22:6)	1.02	0.24	↓
TG(16:0_14:0_16:0)	1.22	5.36	↑	TG(18:3_18:2_18:2)	1.34	0.25	↓
TG(16:0_14:0_16:1)	1.10	2.22	↑	TG(18:2_18:2_18:2)	1.34	0.32	↓
TG(16:0_18:1_20:4)	1.24	0.57	↓	TG(20:0_18:1_18:1)	1.42	0.39	↓
TG(17:0_18:1_18:1)	1.31	0.60	↓	TG(16:1_16:1_18:1)	1.42	0.39	↓
TG(18:0_18:0_22:4)	1.13	0.60	↓	TG(15:0_18:2_18:2)	1.38	0.40	↓
TG(19:0_18:1_18:1)	1.29	0.64	↓	TG(16:0_18:2_22:6)	1.01	0.41	↓
TG(19:1_18:1_18:1)	1.32	0.63	↓	TG(16:0_18:2_20:4)	1.20	0.42	↓
TG(18:1_18:1_22:0)	1.31	0.66	↓	TG(18:0_18:0_18:1)	1.35	0.45	↓
TG(15:0_18:1_18:1)	1.12	0.76	↓	TG(20:1_18:1_18:1)	1.40	0.49	↓
TG(18:1_17:1_18:2)	1.43	0.50	↓	TG(18:1_18:1_22:1)	1.38	0.50	↓
TG(18:2_17:1_18:2)	1.16	0.51	↓	TG(16:1_18:1_18:2)	1.34	0.47	↓
TG(16:1_17:1_18:1)	1.46	0.57	↓	DG(18:2_18:2)	1.15	0.46	↓
TG(18:0_18:1_18:1)	1.28	0.57	↓	DG(18:1_18:1)	1.29	0.48	↓
DG(16:0_18:1)	1.24	0.52	↓	DG(16:0_18:2)	1.37	0.49	↓
DG(18:1_18:2)	1.23	0.53	↓	PC(38:5)	1.20	1.56	↑
DG(18:1_20:4)	1.20	0.54	↓	PC(40:6))	1.23	1.57	↑
PC(18:0_20:3)	1.05	0.66	↓	PC(35:2)	1.05	1.56	↑
PC(40:5)	1.21	2.04	↑	PC(35:4)	1.37	1.62	↑
PC(37:5)	1.44	2.47	↑	PC(36:4)	1.36	1.67	↑
SM(d34:1)	1.26	2.05	↑	PC(17:1_20:5)	1.39	1.61	↑
Cer(d18:1_16:0)	1.03	2.17	↑	PC(38:6)	1.45	1.78	↑
TG(17:0_18:1_20:3)	1.15	0.77	↓	PC(34:2)	1.11	1.99	↑
TG(15:0_17:1_17:1)	1.15	1.99	↑	PC(40:7)	1.14	1.46	↑
PE(18:1_22:6)	1.28	1.51	↑	PC(38:4)	1.18	1.49	↑
PE(18:0p_20:4)	1.11	1.58	↑	PI(18:0_20:4)	1.32	1.67	↑
PE(18:1_20:4)	1.30	1.68	↑	SM(d18:1_24:1)	1.11	1.52	↑
PE(16:0_18:2)	1.17	1.76	↑	PE(16:0_22:6)	1.44	1.83	↑
PE(16:0_20:4)	1.34	1.80	↑				

[a] VIP values obtained from OPLS-DA. [b] Fold change (FC) was calculated based on mean ratios for LC5 vs HF. [c] The icons of trend represent increase or reduced levels of lipid species between the two groups.
